# Phenylalanine Plays Important Roles in Regulating the Capacity of Intestinal Immunity, Antioxidants and Apoptosis in Largemouth Bass (*Micropterus salmoides*)

**DOI:** 10.3390/ani13182980

**Published:** 2023-09-20

**Authors:** Changguo Yi, Hualiang Liang, Dongyu Huang, Heng Yu, Chunyu Xue, Jiaze Gu, Xiaoru Chen, Yongli Wang, Mingchun Ren, Lu Zhang

**Affiliations:** 1 Wuxi Fisheries College, Nanjing Agricultural University, Wuxi 214081, China; 2 Key Laboratory of Integrated Rice-Fish Farming Ecology, Ministry of Agriculture and Rural Affairs, Freshwater Fisheries Research Center, Chinese Academy of Fishery Sciences, Wuxi 214081, China; 3 Tongwei Agricultural Development Co., Ltd., Key Laboratory of Nutrition and Healthy Culture of Aquatic, Livestock and Poultry, Ministry of Agriculture and Rural Affairs, Healthy Aquaculture Key Laboratory of Sichuan Province, Chengdu 610093, China

**Keywords:** dietary phenylalanine level, largemouth bass, immunity, antioxidants, apoptosis

## Abstract

**Simple Summary:**

This experiment was conducted to investigate the effects of dietary phenylalanine level on the health of largemouth bass. Juvenile largemouth bass were fed the experimental diet for 8 weeks. In this study, excessive phenylalanine increased the expression of intestinal antioxidant genes in largemouth bass, while phenylalanine deficiency decreased the immune antioxidant capacity. The plasma biochemical results were similar to those of enzyme activity. The expression of protein-metabolism-related genes was significantly increased in the group with higher phenylalanine content. Similarly, the expression of inflammatory factors and apoptotic factors was also significantly increased in the higher phenylalanine group. In conclusion, the imbalance of phenylalanine in the diet could lead to the decrease of intestinal immunity and antioxidant capacity, and the increase of intestinal cell apoptosis.

**Abstract:**

This experiment was planned to explore the role of dietary phenylalanine levels in intestinal immunity, antioxidant activity and apoptosis in largemouth bass (*Micropterus salmoides*). Six iso-nitrogen and iso-energy diets with phenylalanine levels of 1.45% (DPHE1), 1.69% (DPHE2), 1.98% (DPHE3), 2.21% (DPHE4), 2.48% (DPHE5) and 2.76% (DPHE6) were designed. Juvenile largemouth bass were fed the experimental diet for 8 weeks. In this study, the DPHE5 group increased the expression of intestinal antioxidant genes in largemouth bass (*p* < 0.05), and the increase of antioxidant enzyme activities and content of related substances was most concentrated in the DPHE3 and DPHE4 groups (*p* < 0.05). The results of plasma biochemistry were similar to that of enzyme activity. The expression of genes related to the TOR signalling pathway mainly increased significantly in the DPHE5 group (*p* < 0.05). Similarly, the expression of inflammatory factors, as well as apoptotic factors, also showed significant increases in the DPHE5 group (*p* < 0.05). In conclusion, unbalanced phenylalanine in the diet could lead to a decrease in intestinal immune and antioxidant capacity and also cause a decline in the aggravation of intestinal cell apoptosis.

## 1. Introduction

Aquatic products are high-quality protein and unsaturated fatty acid sources that occupy an important part of people’s daily diets [1]. However, the natural supply of wild fish can hardly meet the demand of people, and aquaculture density is also increasing with people’s demand for aquatic products [2]. With the increase in aquaculture density, aquaculture is facing many problems, one of which is the decline in aquatic product immunity [3]. Increasing farming intensification has increased the vulnerability of fish to pathogen damage [4]. We intend to reverse another mistake to solve this problem, which is the abuse of antibiotics and other drugs [5,6]. With the increasing pressure of environmental protection and the limitations of drug use, many medicines used in the past are no longer suitable for aquaculture. To address this issue, nutritional regulation is an important method by which to enhance the immune systems of fish and reduce the risk of disease [7].

Amino acids are key nutrients for immune cells and supply their function. Amino acids that meet the specific needs of immune cells can activate and stimulate the proliferation of immune cells, thus improving biological immunity [8]. As an essential amino acid (EAA), phenylalanine is vital in the growth and development of fish [9]. In addition, phenylalanine is a precursor of tyrosine and plays many important functions in living organisms [10]. It has long been shown that phenylalanine can influence an organism’s immune response [11]. Furthermore, phenylalanine has been shown to alleviate immunosuppression in mice [12]. At the same time, phenylalanine also has a very important effect on the immune capacity of aquatic animals. Previous studies have found that dietary phenylalanine can improve the intestinal barrier health and immune status of juvenile grass carp (*Ctenopharyngodon idella*) [13] and protect the gills against external injuries [14]. In addition, phenylalanine can also enhance the innate immune response in zebrafish (*Danio rerio*) to external bacterial infection and help eradicate antibiotic-resistant bacteria in vivo [15]. Although phenylalanine is important, improper phenylalanine intake can cause damage to the body [16]. However, there are few studies on the effects of phenylalanine on immune function and apoptosis in aquatic animals. Furthermore, there are no studies on the effect of phenylalanine on the enteric immunity and apoptosis of largemouth bass.

The inflammatory response is a vital reaction in the immune process, and various inflammatory factors play a significant function in the body’s inflammatory response [17]. Many investigations have indicated that dietary amino acids can affect the inflammatory response in fish. For example, arginine regulates the inflammatory response in blunt snout bream (*Megalobrama amblycephala*) and rainbow trout (*Oncorhynchus mykiss*) [18,19], valine is able to affect the inflammatory response in tilapia (*Oreochromis niloticus*) [20] and lysine regulates the inflammatory response in largemouth bass (*Micropterus salmoides*) [21]. The role of amino acids in fish immunity is being revealed step by step. In addition, there is evidence that inflammation is closely related to apoptosis, which is an important physiological activity in aquatic animals [22,23]. Past investigations indicated that arginine, methionine and phenylalanine regulate apoptosis in aquatic animals [14,24,25]. Many studies have been conducted on apoptosis in aquaculture [26,27,28]. However, no studies on the effect of phenylalanine nutrition on apoptosis were found for largemouth bass.

As a carnivorous fish, the largemouth bass is a highly valuable global fish species commonly found in China, the United States, Mexico and other places [29,30]. The largemouth bass occupies an important position in the world’s aquaculture species. However, largemouth bass can be easily infected by viruses, which can cause disease [31,32] and huge economic losses. Therefore, there is an urgent need to solve or alleviate the common diseases of largemouth bass in various ways to reduce the corresponding economic losses. The effect of phenylalanine on growth and metabolism has been previously confirmed in largemouth bass, and 1.98% phenylalanine was determined to be beneficial to growth and metabolism [33], which might be closely related to the health status of the body. However, there are no reports on the influence of phenylalanine on immunity in largemouth bass. The influence of phenylalanine intake on the apoptosis gene of largemouth bass needs further study. Hence, the current research aims to explore the influence of phenylalanine on intestinal enzyme activity, intestinal immunity genes and apoptosis genes in largemouth bass.

## 2. Materials and Methods

### 2.1. Experimental Diets

As in our previous study, six isonitrogenous and isoenergetic diets (crude protein 47.28%, energy 14.83 KJ/g) were prepared and supplemented with different levels of phenylalanine (dry matter 1.45% (DPHE1), 1.69% (DPHE2), 1.98% (DPHE3), 2.21% (DPHE4), 2.48% (DPHE5) and 2.76% (DPHE6), respectively) (Table 1) [33]. The selection of supplemental levels was based on the whole-fish phenylalanine content of the largemouth bass [33]. The feed was prepared as follows. All the ingredients were crushed by a shredder until they passed through 80-mesh screens. The treated mixture was then mixed with fish oil and water. SJPS56 × 2 Leavening Machine was used to produce feed with a particle size of 2 mm (Jiangsu Muyang Holdings Co., Ltd., Yangzhou, China). The feed was dried and stored in refrigerator for later use.

### 2.2. Experimental Procedures

The whole culture experiment was conducted at Wuxi Fishery College of Nanjing Agricultural University. Largemouth bass were acclimated to cage conditions for two weeks before the start of the feeding trials. Six experimental groups with three replicates were randomly assigned to eighteen cages (1 cubic metre in size). Twenty juvenile largemouth bass without disease or injury weighing 19.5 g (±0.98 g) were indiscriminately assigned to prepared cages after a day of starvation. Juvenile largemouth bass were fed until apparent satiation at 7:00 and 17:00 daily. The number of dead fish was recorded every day during the eight-week experiment. All fish were weighed every two weeks to record weight changes and adjust feeding. During the experiment, the temperature of the water was 28–30 °C, the dissolved oxygen in the cages was more than 6 mg/L, and the pH of culture cycle was maintained at 7.0–7.8.

### 2.3. Sample Collection and Experimental Treatment

After 8 weeks of feeding experiments, all largemouth bass fasted for a day before sampling to empty their intestines. The fish were anaesthetized with MS-222 prior to sampling, after which the intestines of the largemouth bass were collected and stored in liquid nitrogen in cryopreservation tubes. After that, the samples were transferred to a −80 °C refrigerator. The intestinal tissue was homogenized with normal saline at a ratio of 1:9 and then centrifuged. After that, the supernatant was stored in a −80 °C refrigerator for subsequent analysis and determination. Intestinal RNA was extracted with TRIzol (Nanjing Vazyme BioTech Co., Ltd., Nanjing, China). As such, the tissue was homogenized in TRIzol, 1/5 volume of chloroform was added and then the sample was vigorously shaken and left to sit for five minutes. The supernatant was taken after centrifugation, and the same amount of isopropanol was added and left to stand for 10 min. Then, the supernatant was discarded after the sample was centrifuged. After washing the precipitate with 75% ethanol, it was centrifuged; then, the supernatant was discarded, and the precipitate was dried and dissolved in DEPC water. Then, the samples were transferred to a −80 °C refrigerator.

### 2.4. Laboratory Determination

The intestinal enzyme activity of largemouth bass was determined by a kit. The malondialdehyde (MDA), total protein (TP), glutathione (GSH), catalase (CAT), total antioxidant capacity (T-AOC) and total superoxide dismutase (T-SOD) were all from NanJing JianCheng Bioengineering Institute (Nanjing, China).

Plasma biochemistry was analysed by an Mindray BS-400 automatic analyser (Shenzhen, China). Alkaline phosphatase (ALP), aminotransferase (ALT), high-density lipoprotein (HDL-C), albumin (ALB), low-density lipoprotein (LDL-C) and alanine aspartate aminotransferase (AST) kits were all from Shanghai Zhicheng Biological Technology Co., Ltd. (Shanghai, China).

The relative expression of intestinal genes was detected by fluorescence quantitative PCR (qPCR). A One-Step qRT–PCR SYBR Green Kit (Nanjing Vazyme BioTech Co., Ltd., Nanjing, China) was used for qPCR on a 7500 fluorescence quantitative PCR machine. Table 2 shows the specific primers used in this experiment. Beta-actin (β-actin) was selected as a nonregulatory internal reference gene. The relative expressions were analysed by a relative standard curve method.

### 2.5. Statistical Analysis

SPSS 25.0 software was used to analyse all experimental data with the one-way ANOVA and Tukey’s multiple comparison tests. The difference between groups was statistically significant when *p* < 0.05. Groups with significant differences were represented by different letters.

## 3. Results

### 3.1. Intestinal Antioxidant Indices and Plasma Biochemical Indices

The enzyme activity of intestinal tissue is shown in Table 3. In comparison to the DPHE1 group, the T-SOD activity of the DPHE3 group was significantly increased, while significantly lower T-SOD activity was found in the DPHE6 group compared with the DPHE4 group (*p* < 0.05). Except for the DPHE3 group, the content of MDA was significantly decreased in the DPHE2 group. In comparison to the DPHE1 group, the content of GSH was significantly increased in the DPHE3 group and peaked in the DPHE4 group (*p* < 0.05). The activity of T-AOC was the highest in the DPHE4 group, but no significant difference was found compared with the DPHE1 group (*p* > 0.05). CAT activity increased with increasing phenylalanine content, and the DPHE6 group had significantly more CAT activity than the DPHE1 group (*p* < 0.05).

The results of plasma biochemistry are shown in Table 4. Significantly higher ALP was found in the DPHE2, DPHE3 and DPHE5 groups compared with the DPHE1 group (*p* < 0.05). In addition, significantly lower ALB was found in the DPHE1 group compared with other groups (*p* < 0.05). No significant difference was found in LDL-C (*p* < 0.05), and significantly higher HDL-C was found in the DPHE2, DPHE 3, DPHE 4 and DPHE6 groups compared with the DPHE1 group (*p* < 0.05). The levels of ALT and AST in the DPHE4 group were prominently higher than those of the DPHE1 group (*p* < 0.05).

### 3.2. Intestinal Gene Expression of the TOR Pathway

**Figure 1:**
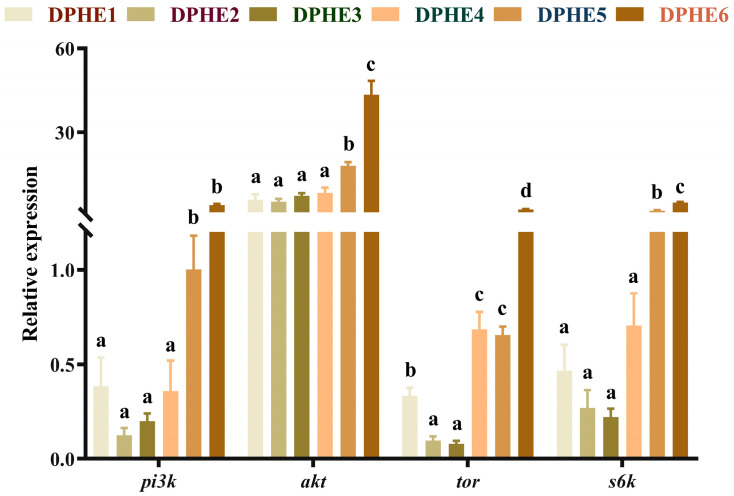
Relative mRNA expression of *TOR*-signalling-pathway-related genes in the intestinal tract of largemouth bass. Phosphatidylinositol 3-kinase, *pi3k*; protein kinase B, *akt*; target of rapamycin, *tor*; ribosomal protein S6 kinase-polypeptide, *s6k*. ^a–d^ Different groups with significant differences are represented by different letters, different groups without significant differences are represented by the same letter, and no letter means that there is no significant difference between all groups.

The expression results of the target of rapamycin (TOR) signalling pathway are shown in Figure 1. Significantly higher mRNA levels of phosphatidylinositol 3-kinase (*pi3k*), protein kinase B (*akt*) and ribosomal protein S6 kinase-polypeptide (*s6k*) were found in the DPHE5 group compared with those in the lower experimental groups (DPHE1, DPHE2, DPHE3 and DPHE4). The mRNA level reached the highest expression in the DPHE6 group and significantly higher mRNA levels were found in it compared with the other phenylalanine groups (*p* < 0.05). The mRNA expression of *tor* in the DPHE2 group and DPHE3 group was prominently decreased compared with that in the DPHE1 group (*p* < 0.05). Significantly higher mRNA levels of *tor* were found in the DPHE4 group and DPHE5 group, and reached the highest level in the DPHE6 group (*p* < 0.05), compared with the DPHE1 group (*p* < 0.05).

### 3.3. The Expression Results of Immune-Related Genes

**Figure 2:**
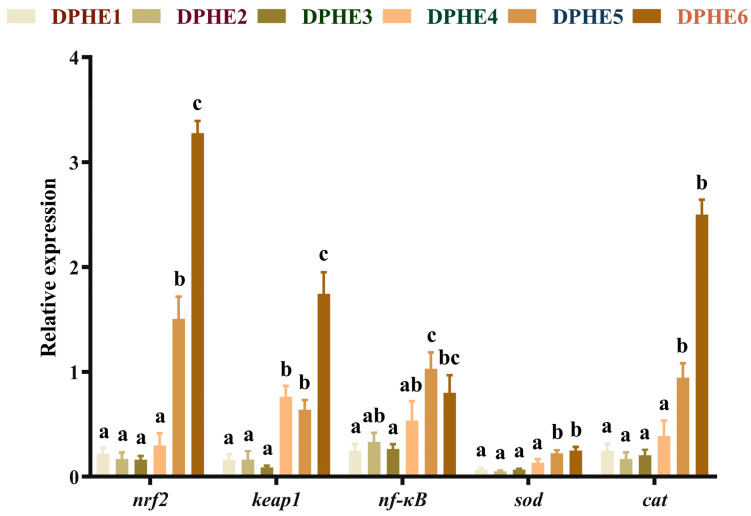
Relative mRNA expression of intestinal immune antioxidant genes in largemouth bass. Nuclear factor erythroid2-related factor 2, *nrf2*; Kelch-like ECH-associated protein 1, *keap1*; nuclear factor kappa-B, *nf-κB*; superoxide dismutase, *sod*; catalase, *cat*. ^a–c^ Different groups with significant differences are represented by different letters, different groups without significant differences are represented by the same letter, and no letter means that there is no significant difference between all groups.

The results of immune-related genes are shown in Figure 2. Significantly higher mRNA level of nuclear factor erythroid2-related Factor 2 (*nrf2*) was found in the DPHE5 group in comparison with the DPHE1 group (*p* < 0.05) and attained the highest expression in the DPHE6 group (*p* < 0.05). Prominently higher mRNA levels of Kelch-like ECH-associated protein 1 (*keap1*) were found in the DPHE4 group and DPHE5 group in comparison to the DPHE1 group (*p* < 0.05). The mRNA levels of nuclear factor kappa-B (*nf-κb*) in the DPHE5 group was the highest and significantly greater than that in the DPHE1 group (*p* < 0.05). The mRNA levels of superoxide dismutase (*sod*) decreased first and then increased, with a significant elevation in the DPHE5 group (*p* < 0.05); they showed the greatest expression in the DPHE6 group (*p* < 0.05). Significantly higher expression of catalase (*cat*) mRNA in the DPHE5 group was found by comparison with the DPHE1 group (*p* < 0.05).

### 3.4. The Expression Results of Inflammatory Factors

**Figure 3:**
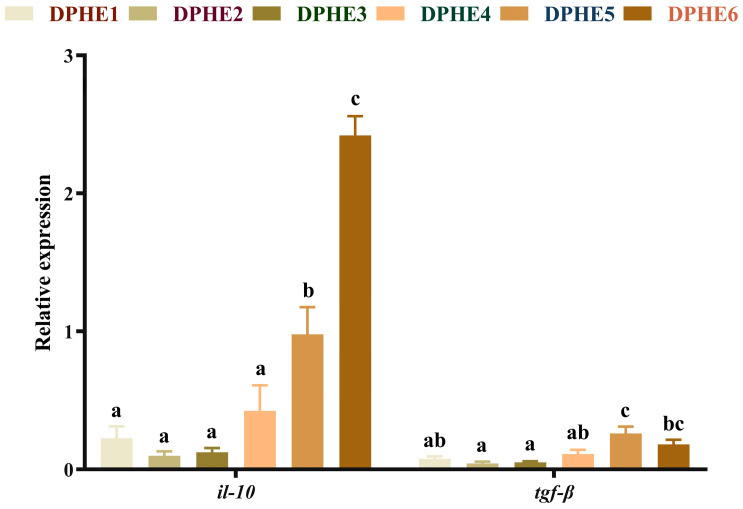
Relative mRNA expression of intestinal anti-inflammatory factor genes in largemouth bass. Interleukin-10, *il-10*; transforming growth factor-β, *tgf-β*. ^a–c^ Different groups with significant differences are represented by different letters, different groups without significant differences are represented by the same letter, and no letter means that there is no significant difference between all groups.

The expression results of anti-inflammatory factors are shown in Figure 3. A significantly higher mRNA level of interleukin-10 (*il-10*) was found in the DPHE5 group and the mRNA level reached the highest expression level in the DPHE6 group (*p* < 0.05). The highest mRNA level of transforming growth factor-β (*tgf-β*) was found in the DPHE5 group, which was also significantly elevated compared with the DPHE1 group (*p* < 0.05).

**Figure 4:**
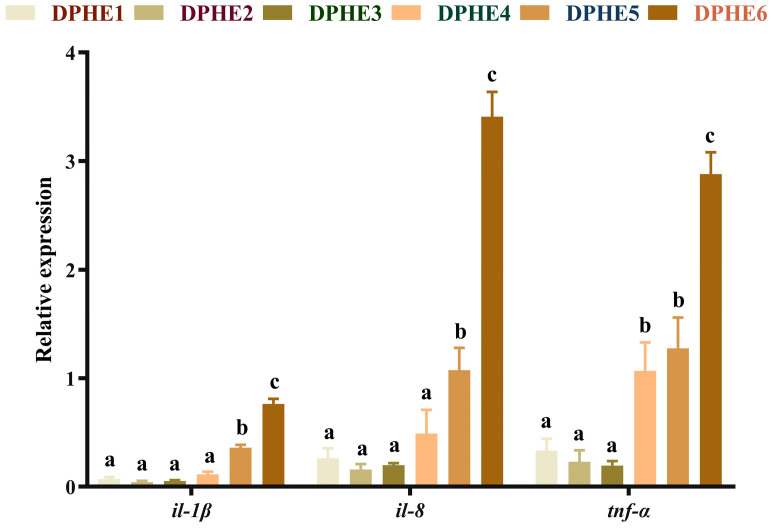
Relative mRNA expression of intestinal proinflammatory factor genes in largemouth bass. Interleukin-1β, *il-1β*; interleukin-8, *il-8*; tumour necrosis factor-α, *tnf-α*. ^a–c^ Different groups with significant differences are represented by different letters, different groups without significant differences are represented by the same letter, and no letter means that there is no significant difference between all groups.

The expression results of proinflammatory factors are shown in Figure 4. Significantly higher mRNA levels of interleukin-1β (*il-1β*) and interleukin-8 (*il-8*) were found in the DPHE5 group compared with the DPHE1 group and the highest mRNA expression was reached in the DPHE6 group (*p* < 0.05). The mRNA levels of tumour necrosis factor-α (*tnf-α*) were significantly improved in the DPHE4 group.

### 3.5. The Expression Results of Apoptosis-Related Genes

**Figure 5:**
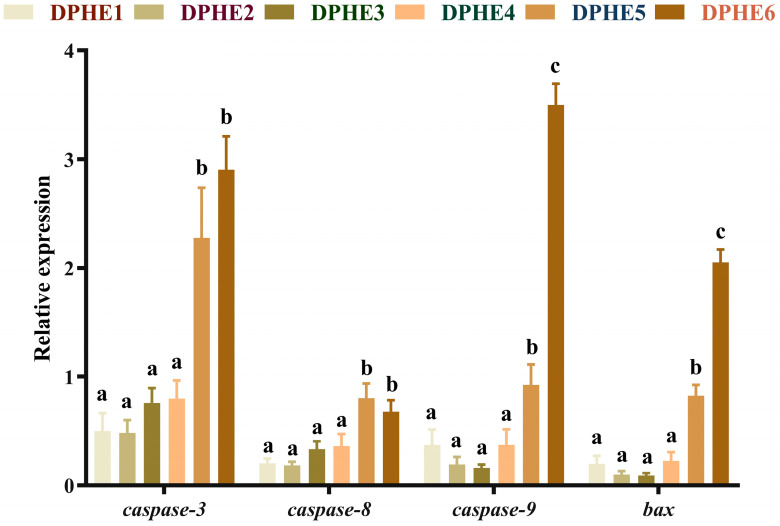
Relative mRNA expression of intestinal pro-apoptosis-related genes in largemouth bass. Cysteinyl aspartate specific proteinase 3, *caspase-3*; cysteinyl aspartate specific proteinase 8, *caspase-8*; cysteinyl aspartate specific proteinase 9, *caspase-9*; BCL2-Associated X, *bax*. ^a–c^ Different groups with significant differences are represented by different letters, different groups without significant differences are represented by the same letter, and no letter means that there is no significant difference between all groups.

**Figure 6:**
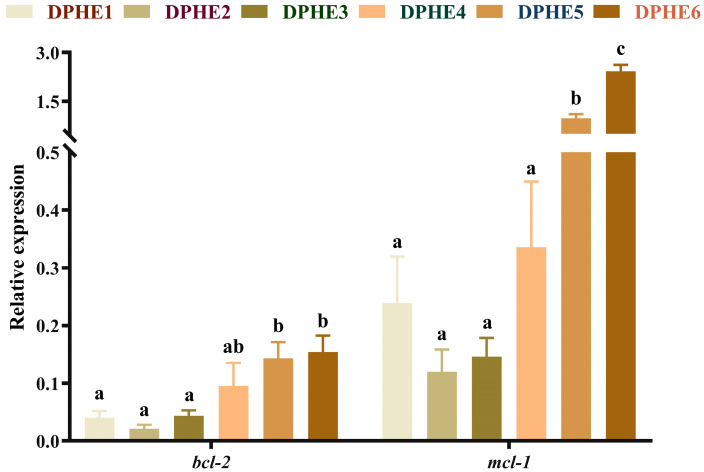
Relative mRNA expression of intestinal anti-apoptosis-related genes in largemouth bass. B-cell lymphoma-2, *bcl-2*; myeloid cell leukemia-1, *mcl-1*. ^a–c^ Different groups with significant differences are represented by different letters, different groups without significant differences are represented by the same letter, and no letter means that there is no significant difference between all groups.

The expression results of apoptosis-related genes are shown in Figure 5 and Figure 6. In terms of apoptosis, significantly higher mRNA levels of bcl2-associated X (*bax*), cysteinyl aspartate-specific proteinase 3 (*caspase-3*), cysteinyl aspartate-specific proteinase 8 (*caspase-8*) and cysteinyl aspartate-specific proteinase 9 (*caspase-9*) were found in the DPHE5 group and DPHE6 group compared with the DPHE1 group (*p* < 0.05). Myeloid cell leukaemia-1 (*mcl-1*) and B-cell lymphoma-2 (*bcl-2*) mRNA levels first decreased and then increased, and they were significantly increased in the DPHE5 group and DPHE6 group (*p* < 0.05).

## 4. Discussion

It is known that the intake of nutrients affects not only the growth and development but also the health of fish. For juvenile fish, unbalanced nutrient intake will affect their health and may even lead to the occurrence of diseases [37]. The current research has shown that phenylalanine was equally strongly linked to the health of largemouth bass. Plasma biochemical indices are often used as one of the criteria to judge fish health [38,39]. In the current study, the results made it clear that dietary phenylalanine deficiency led to a decrease in ALP, ALB and HDL-C, suggesting that dietary phenylalanine deficiency may adversely affect the health of largemouth bass. This is similar to the findings on grass carp [40]. Otherwise, in the current experiment, the liver function indices, such as ALT and AST, demonstrated a pattern of first increasing and then decreasing and reached the highest value in the DPHE4 experimental group. The authors speculate that this may be due to vigorous metabolism rather than liver damage [33] because AST and ALT also play an important role in fish nutrient metabolism [41]. In the present experiment, no significant change was found in LDL-C. Other amino acids have been shown to do the same for fish in past studies [42].

In this study, both deficient (DPHE1) and excess (DPHE4, DPHE5 and DPHE6) phenylalanine diets showed higher levels of MDA. MDA can indicate cellular oxidative stress as a measure of oxidative stress, and high levels of MDA indicate that the imbalance of phenylalanine may lead to oxidative damage [43]. The same is true of other amino acids and fish [44,45]. At the same time, unbalanced dietary phenylalanine levels decreased the activity of T-SOD, T-AOC and the content of GSH in the current study. Because antioxidant enzymes are crucial for avoiding oxidative stress in fish and scavenging superoxide anions and hydroxyl radicals [45], the increase in MDA may be clearly related to the decline in antioxidant enzyme activity. Dietary phenylalanine deficiency caused a decrease in T-SOD in largemouth bass similar to other amino acids [44]. However, the activity of CAT continued to increase in this study. This outcome is slightly different from that found in a study of grass carp and tilapia [40,46]. Past research has shown that CAT activity is affected by *nrf2* expression [47]. Although the specific mechanism is not clear, the results were similar to the simultaneous increase in CAT activity and *nrf2* expression found in this experiment. In addition, the simultaneous increase in *nrf2* and *keap1* caused by excessive dietary phenylalanine indicates that the fish’s body may perform feedback regulation in response to the decrease in the body’s antioxidant capacity [48]. In the current study, dietary phenylalanine deficiency (DPHE1) had no significant effect on the mRNA expression of *nrf2* and *keap1*. However, higher dietary phenylalanine (DPHE5 and DPHE6) significantly increased the relative mRNA expression levels of *nrf2* and *keap1* and then increased the relative mRNA expression levels of the downstream genes *sod* and *cat*. The authors speculate that this may be due to the regulatory performance of the body in response to the production of inflammation and the decrease in immune antioxidant enzyme activity [48]. Additionally, significantly higher mRNA levels of *pi3k*, *akt* and *s6k* upstream and downstream of *tor* were found in the DPHE5 experimental group, but no significant difference was shown in low phenylalanine groups. As for the upstream genes of *nrf2* and *keap1*, the increased expression of *pi3k* and *akt* may be one of the reasons for the increased expression of the former [49]. Therefore, excessive dietary phenylalanine could negatively affect the immune antioxidant capacity of largemouth bass. This is consistent with previous findings that excessive phenylalanine intake can suppress immunity in fish [13].

The inflammatory response is another important measure of aquatic animal health [50], and inflammatory cytokines are regulated by the *tor* signalling pathway [51]. In the current study, the mRNA levels of *tor* in the DPHE1 group and DPHE4 group were significantly higher than the mRNA levels of *tor* in the DPHE2 group and DPHE3 group. Interestingly, in a study of grass carp, the opposite result was observed [13]. This altered gene expression appears to be associated with an unbalanced dietary phenylalanine diet, and its underlying causes need further investigation. The inflammatory response is in turn regulated by upstream *nf-κb* [52]. In this study, DPHE1 showed no significant effect on the expression of *nf-κb*, while DPHE5 showed an increase in *nf-κb* expression, which resulted in an increase in the downstream proinflammatory factors *tnf-α*, *il-1β* and *il-8*. Excessive dietary phenylalanine has shown similar results in previous grass carp studies [13]. Interestingly, previous research in grass carp has found that phenylalanine deficiency causes an upregulation of proinflammatory factors [13]. This may be due to the different tolerances of amino acid deficiency in different fish [9,53]. In addition, in this study, the anti-inflammatory factors *il-10* and *tgf-β* were also highly expressed under the influence of excessive dietary phenylalanine. This may be due to a negative feedback mechanism in the body that leads to increased expression of both anti-inflammatory and proinflammatory factors [54]. In addition to acting as an anti-inflammatory factor, *tgf-β* is thought to regulate inflammation and work with *il-10* to regulate inflammation, according to previous research [55]. The authors speculate that the body expresses many anti-inflammatory factors in response to the inflammation caused by phenylalanine. In summary, DPHE5 and DPHE6 were found to induce an inflammatory response in largemouth bass.

Cell apoptosis is closely related to the caspase family and bcl-2 family [56,57]. *Caspase-3*, *caspase-8*, *caspase-9* and *bax* are all proapoptotic genes. In the present study, significantly higher mRNA levels of proapoptotic genes in the DPHE5 group and DPHE6 group were found compared with the expression levels in the other groups. This seemed to imply that experimental groups DPHE5 and DPHE6 induced apoptosis in the intestinal cells of largemouth bass but experimental group DPHE1 had a limited effect on apoptosis. Similar to the findings of a previous study, the accumulation of phenylalanine in organisms was found to exacerbate nerve cell apoptosis [58]. Interestingly, previous research on grass carp gills has indicated that phenylalanine deficiency exacerbates apoptosis [14]. The reason for this dissimilarity may be due to the species of organisms and discrepancies in biological organization. In addition, in the current study, *bcl-2* and *mcl-1* were also highly expressed in the DPHE5 experimental group. A previous study suggested that *nf-κb* could alleviate apoptosis by activating *bcl-2* [59]. Therefore, it is reasonable to assume that the activation of *nf-κb* might be an important cause of the increased expression of *bcl-2* and *mcl-1*. Taken together, in this study, 2.48% or more dietary phenylalanine resulted in the increased apoptosis of intestinal cells in largemouth bass.

## 5. Conclusions

Low or high levels of dietary phenylalanine (1.45% or 2.48%) can lead to a decline in the intestinal immune and antioxidant capacity of largemouth bass, while high levels of dietary phenylalanine can also lead to the aggravation of intestinal cell apoptosis in largemouth bass. In this experiment, dietary supplementation with 1.98–2.21% (4.19–4.67% dietary protein) phenylalanine was beneficial to the intestinal health of largemouth bass. Compared with previous studies, this study demonstrated the effect of phenylalanine level on the health of largemouth bass, provided guidance for the addition of phenylalanine in aquafeed to reduce fish health loss and cost increase caused by unbalanced amino acids and provided certain help for subsequent production research.

## Figures and Tables

**Table 1:** Formulation and proximate composition of the experimental diets (% dry matter).

Ingredients (%)	
Fish meal ^a^	25
Rapeseed meal ^a^	8
Soybean meal ^a^	6
Corn gluten meal ^a^	8
Wheat flour ^a^	16
Rice bran	8
Fish oil	5
Sleeve-Fish Ointment	2
Amino acid premix ^b^	13.02
Choline chloride	0.1
Vitamin premix ^c^	1
Mineral premix ^d^	1
Calcium dihydrogen phosphate	1
Microcrystalline cellulose	4.57
Ethoxy quinoline	0.01
Glycine	*
Phenylalanine	**
Vitamin C	0.05
Total	100
Actual phenylalanine level	***
Actual tyrosine level	1.32–1.34

The feed formulation references Yi et al. [33]. * means increased additive glycine levels (1.25, 1, 0.75, 0.5, 0.25 and 0%, respectively), ** means increased additive phenylalanine levels (0, 0.25, 0.5, 0.75, 1 and 1.25%, respectively), *** means actual phenylalanine levels (1.45, 1.69, 1.98, 2.21, 2.48 and 2.76%, respectively) of six diets. ^a^ All ingredients were obtained from Wuxi Tongwei feedstuffs Co., Ltd. (Wuxi, China); fish meal, crude protein 656 g/kg, crude lipid 94.6 g/kg; rapeseed meal, crude protein 37.5%, crude lipid 1.4%; soybean meal, crude protein 507.9 g/kg, crude lipid 42.5 g/kg; corn gluten meal, crude protein 55.9%, crude lipid 3.3%; wheat flour, crude protein 11.8%, crude lipid 1.2%. ^b^ Amino acid premix (g/100 g of premix): Arginine, 1.06; Histidine, 0.45; Isoleucine, 0.77; Leucine, 0.80; Lysine, 1.69; Methionine, 0.36; Threonine, 0.79; Valine, 0.77; Tryptophan, 0.18; Aspartic acid, 0.47; Serine, 0.68; Glycine, 0.90; Alanine, 1.36; Glutamic acid, 1.95; Proline, 0.8. ^c^ Vitamin premix (IU or mg/kg of premix): vitamin A, 800,000 IU; vitamin D3, 150,000–250,000 IU; vitamin E, 4500 IU; vitamin K3, 600 mg; thiamin, 800 mg; riboflavin, 800 mg; calcium pantothenate, 2000 mg; pyridoxine HCl, 2500 mg; cyanocobalamin, 8 mg; biotin, 16 mg; folic acid, 400 mg; niacin, 2800 mg; inositol, 10,000 mg; vitamin C, 10,000 mg. ^d^ Mineral premix (g/kg of premix): calcium biphosphate, 20 g; sodium chloride, 2.6 g; potassium chloride, 5 g; magnesium sulphate, 2 g; ferrous sulphate, 0.9 g; zinc sulphate, 0.06 g; cupric sulphate, 0.02; manganese sulphate, 0.03 g; sodium selenate, 0.02 g; cobalt chloride, 0.05 g; potassium iodide, 0.004 g; and zeolite was used as a carrier.

**Table 2:** Primer sequences for qPCR.

Gene	Forward Sequence (5′–3′)	Reverse Sequence (5′–3′)	Accession Number/Reference
*pi3k*	CTCACCATGGAGGATGGACC	ACGGTGGGAGTGGAGGTTTA	Cluster-21914.23096
*akt*	AGCGCACCTTCCATGTAGAC	GGCTATTTGCCACTTGCTGG	AXE72881.1
*tor*	CCATCCTCAACCTACTTCC	CTCTCCTTCTCCTTCTTCAG	Cluster-21914.16479
*s6k*	GTAATGCAAAGGACACGGCG	GTTCCCCACCGCTCAGATAC	XP_010747297.3
*keap1*	CGTACGTCCAGGCCTTACTC	TGACGGAAATAACCCCCTGC	Cluster-21914.26115
*nrf2*	AGAGACATTCGCCGTAGA	TCGCAGTAGAGCAATCCT	NM_212855.2
*nf-κB*	CCACTCAGGTGTTGGAGCTT	TCCAGAGCACGACACACTTC	Cluster-21914.7253
*il-10*	CGGCACAGAAATCCCAGAGC	CAGCAGGCTCACAAAATAAACATCT	Yang et al., 2020 [21]
*tgf-β*	GCTCAAAGAGAGCGAGGATG	TCCTCTACCATTCGCAATCC	[34]
*il-1β*	CGTGACTGACAGCAAAAAGAGG	GATGCCCAGAGCCACAGTTC	[34]
*tnf-α*	CTTCGTCTACAGCCAGGCATCG	TTTGGCACACCGACCTCACC	[34]
*il-8*	TCGGTCCTCCTGGGTGAAAA	GTGCTCCTTCCTGCTGATGTA	Cluster-21914.20189
*sod*	TGGCAAGAACAAGAACCACA	CCTCTGATTTCTCCTGTCACC	[35]
*cat*	CTATGGCTCTCACACCTTC	TCCTCTACTGGCAGATTCT	MK614708.1
*caspase-3s*	GAGGCGATGGACAAGAGTCA	CACAGACGAATGAAGCGTGG	XM_038713063.1
*caspase-8*	GAGACAGACAGCAGACAACCA	TTCCATTTCAGCAAACACATC	[36]
*caspase-9*	CTGGAATGCCTTCAGGAGACGGG	GGGAGGGGCAAGACAACAGGGTG	[36]
*bax*	ACTTTGGATTACCTGCGGGA	TGCCAGAAATCAGGAGCAGA	[36]
*bcl-2*	CCAACGTCATGGTTGTCATGG	GTGGAGCCAACCAGGAATCT	Cluster-21914.31403
*mcl-1*	GTGGCCAACAATGAGAAGGC	AGGAGTCTCTGTTCGTCCGT	Cluster-21914.26326
*β-actin*	ATGCAGAAGGAGATCACAGCCT	AGTATTTACGCTCAGGTGGGG	AF253319.1

*pi3k*, phosphatidylinositol 3-kinase; *akt*, protein kinase B; *tor*, target of rapamycin; *s6k*, ribosomal protein S6 kinase-polypeptide; *keap1*, Kelch-like ECH-associated protein 1; *nrf2*, nuclear factor erythroid2-related factor 2; *nf*-*κB*, nuclear factor kappa-B; *il-10*, interleukin-10; *tgf-β*, transforming growth factor-β; *il-1β*, interleukin-1β; *tnf-α*, tumor necrosis factor-α; *il-8*, interleukin-8; *sod*, superoxide dismutase; *cat,* catalase; *caspase-3*, cysteinyl aspartate specific proteinase 3; *caspase-8*, cysteinyl aspartate specific proteinase 8; *caspase-9*, cysteinyl aspartate specific proteinase 9; *bax*, Bcl2-associated X; *bcl-2*, B-cell lymphoma-2; *mcl-1*, myeloid cell leukemia-1; *β-actin*, beta-actin.

**Table 3:** Activity and content of total superoxide dismutase (T-SOD), malondialdehyde (MDA), glutathione (GSH), total antioxidant capacity (T-AOC) and catalase (CAT) in intestines of largemouth bass after 8 weeks of feeding experimental diet ^1^.

Parameters	Dietary Phenylalanine Group					
	DPHE1	DPHE2	DPHE3	DPHE4	DPHE5	DPHE6
T-SOD ^2^ (U/mgprot)	4.75 ± 0.18 ^a^	5.79 ± 0.12 ^ab^	6.77 ± 0.10 ^bc^	7.29 ± 0.38 ^c^	6.46 ± 0.94 ^bc^	5.38 ± 0.66 ^ab^
MDA ^2^ (nmol/mgprot)	6.40 ± 0.94 ^b^	4.17 ± 0.16 ^a^	5.10 ± 0.60 ^ab^	6.68 ± 0.47 ^b^	6.99 ± 0.32 ^b^	7.18 ± 0.28 ^b^
GSH ^2^ (umol/gprot)	41.19 ± 2.59 ^a^	35.00 ± 2.21 ^a^	58.09 ± 4.22 ^bc^	80.84 ± 2.23 ^d^	67.21 ± 5.34 ^c^	46.92 ± 4.77 ^ab^
T-AOC ^2^ (mmol/g)	0.66 ± 0.05 ^ab^	0.75 ± 0.08 ^ab^	0.72 ± 0.03 ^ab^	0.80 ± 0.04 ^b^	0.68 ± 0.03 ^ab^	0.59 ± 0.03 ^a^
CAT ^2^ (U/mgprot)	74.78 ± 11.12 ^a^	81.27 ± 9.35 ^ab^	96.82 ± 9.12 ^ab^	98.21 ± 17.01 ^ab^	98.49 ± 13.68 ^ab^	130.41 ± 13.91 ^b^

^1^ The difference of superscript on the same line is statistically significant with different letters (*p* < 0.05), while no superscript indicates no significant difference (*p* > 0.05). ^2^ Superoxide dismutase (T-SOD), malondialdehyde (MDA), glutathione (GSH), total antioxidant capacity (T-AOC), catalase (CAT).

**Table 4:** Plasma alkaline phosphatase (ALP), albumin (ALB), alanine aminotransferase (ALT), aspartate aminotransferase (AST), low-density lipoprotein (LDL-C) and high-density lipoprotein (HDL-C) of largemouth bass after 8 weeks of feeding experimental diet ^1^.

Parameters	Dietary Phenylalanine Group					
	DPHE1	DPHE2	DPHE3	DPHE4	DPHE5	DPHE6
ALP ^2^ (U/L)	23.36 ± 1.49 ^a^	41.06 ± 3.68 ^b^	37.26 ± 2.51 ^b^	34.81 ± 4.00 ^ab^	37.50 ± 4.52 ^b^	27.32 ± 1.93 ^ab^
ALB ^2^ (g/L)	9.47 ± 0.40 ^a^	12.88 ± 0.44 ^b^	13.94 ± 1.14 ^b^	12.71 ± 0.58 ^b^	12.06 ± 0.76 ^b^	13.46 ± 0.60 ^b^
ALT ^2^ (U/L)	0.83 ± 0.16 ^a^	1.03 ± 0.20 ^ab^	1.14 ± 0.20 ^ab^	1.81 ± 0.27 ^b^	1.37 ± 0.28 ^ab^	1.08 ± 0.17 ^ab^
AST ^2^ (U/L)	18.38 ± 2.05 ^a^	21.18 ± 1.73 ^a^	22.48 ± 1.13 ^a^	30.02 ± 0.99 ^b^	23.02 ± 1.58 ^a^	19.93 ± 1.58 ^a^
LDL-C ^2^ (mmol/L)	0.94 ± 0.13 ^a^	0.93 ± 0.05 ^a^	1.00 ± 0.10 ^a^	0.92 ± 0.07 ^a^	0.91 ± 0.06 ^a^	1.15 ± 0.06 ^a^
HDL-C ^2^ (mmol/L)	2.49 ± 0.11 ^a^	3.62 ± 0.13 ^c^	3.66 ± 0.13 ^c^	3.36 ± 0.15 ^bc^	2.92 ± 0.13 ^ab^	3.59 ± 0.16 ^c^

^1^ The difference of superscript on the same line is statistically significant with different letters (*p* < 0.05), while no superscript indicates no significant difference (*p* > 0.05). ^2^ Alkaline phosphatase (ALP), albumin (ALB), alanine aminotransferase (ALT), aspartate aminotransferase (AST), low-density lipoprotein (LDL-C), high-density lipoprotein (HDL-C).

## Data Availability

The authors confirm that the data supporting the findings of this study are available within the manuscript, figures and tables.
